# Supplementary material for: Single-Cell Analyses of a Novel Mouse Urothelial Carcinoma Model Reveal a Role of Tumor-Associated Macrophages in Response to Anti-PD-1 Therapy
Source: Cancers (Basel). 2022 May 19;14(10):2511. doi: 10.3390/cancers14102511 (PMC9139541; doi:10.3390/cancers14102511)
Supplement: Supplementary file 1 [file cancers-14-02511-s001.zip › Supplementary data.pdf]

Supplementary Materials

Single-Cell Analyses of a Novel Mouse Urothelial Carcinoma Model Reveal a Role of Tumor-Associated Macrophages in Response to Anti-PD-1 Therapy

Dongbo Xu [1], Li Wang [1], Kyle Wieczorek [1], Yali Zhang [2], Zinian Wang [3], Jianmin Wang [2], Bo Xu [4], Prashant K Singh [5], Yanqing Wang [6], Xiaojing Zhang [6], Yue Wu [1], Gary J. Smith [1], Kristopher Attwood [2], Yuesheng Zhang [6], David W. Goodrich [6], Qiang Li [1, 6, *]

[1] Department of Urology, Roswell Park Comprehensive Cancer Center, Buffalo, NY 14203, USA; Dongbo.Xu@RoswellPark.org (D.X.); Li.Wang@RoswellPark.org (L.W.); Kyle.Wieczorek@RoswellPark.org (K.W.); Yue.Wu@RoswellPark.org (Y.W.); Gary.Smith@RoswellPark.org (G.J.S.)

[2] Department of Biostatistics & Bioinformatics, Roswell Park Comprehensive Cancer Center, Buffalo, NY 14203, USA; Yali.Zhang@RoswellPark.org (Y.Z.); Jianmin.Wang@RoswellPark.org (J.W.); Kristopher.Attwood@RoswellPark.org (K.A.)

[3] Departments of Cancer Prevention and Control, Roswell Park Comprehensive Cancer Center, Buffalo, NY 14203, USA; Zinian.Wang@RoswellPark.org

[4] Departments of Pathology, Roswell Park Comprehensive Cancer Center, Buffalo, NY 14203, USA; Bo.Xu@RoswellPark.org

[5] Departments of Cancer Genetics & Genomics, Roswell Park Comprehensive Cancer Center, Buffalo, NY 14203, USA; Prashant.Singh@RoswellPark.org

[6] Department of Pharmacology & Therapeutics, Roswell Park Comprehensive Cancer Center, Buffalo, NY 14203, USA; Yanqing.Wang@RoswellPark.org (Y.W.); Xiaojing.Zhang@RoswellPark.org (X.Z.); Yuesheng.Zhang@RoswellPark.org (Y.Z.); David.Goodrich@RoswellPark.org (D.W.G.)

* Correspondence: Qiang.Li@RoswellPark.org; Tel.: +1-716-845-3389; Fax: +1-716-845-3300

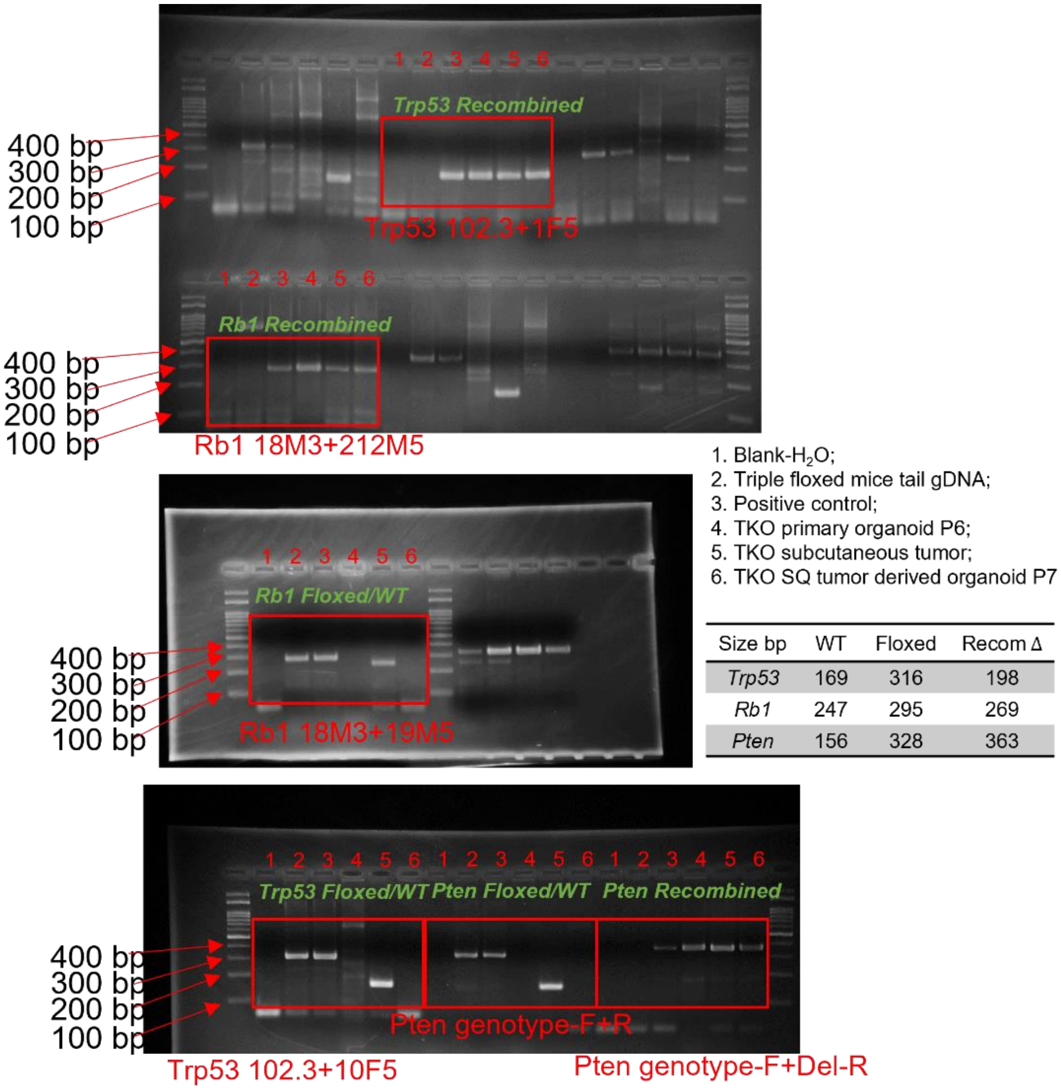

1. Blank-H_2O;
2. Triple floxed mice tail gDNA;
3. Positive control;
4. TKO primary organoid P6;
5. TKO subcutaneous tumor;
6. TKO SQ tumor derived organoid P7

Size bp	WT	Floxed	Recom Δ
Trp53	169	316	198
Rb1	247	295	269
Pten	156	328	363

Supplemental Figure S1. The original PCR results using DNA gel electrophoresis. Gels were stained with ethidium bromide. The expected size of PCR bands (WT, Floxed, and Recombination alleles) were listed in the table. Sequences of primers in red fonts were listed in Supplemental Table S2.

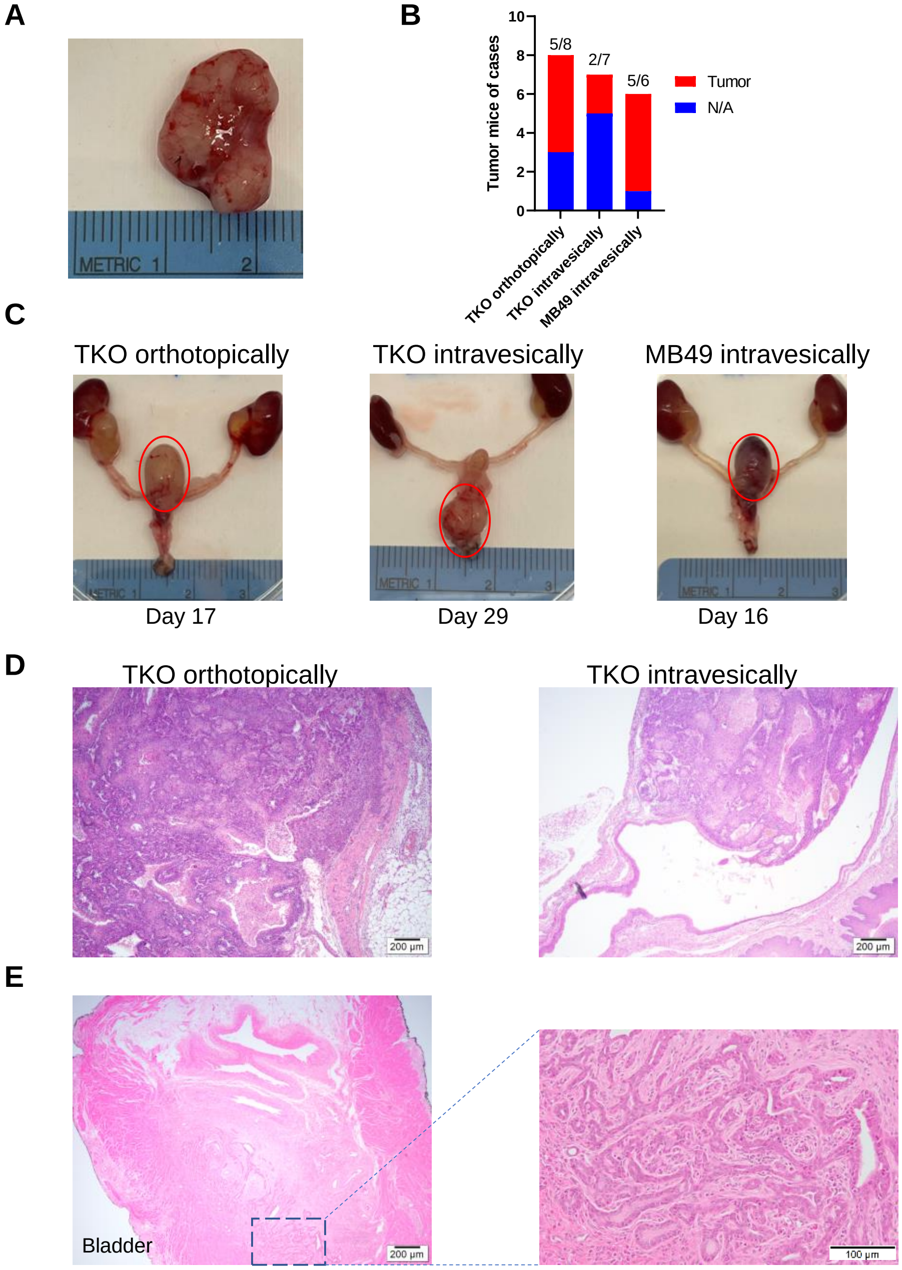

Supplemental Figure S2. The tumor formation of TKO cells by subcutaneous, orthotopic and intravesical injections in C57 BL/6J mice. (**A**) A gross TKO subcutaneous tumor on day 14 was shown after subcutaneous injection of 2 million TKO cell suspensions. (**B**) Tumors take rates of 2 million TKO cell suspension injection orthotopically and intravesically. (**C**) Representative gross pictures of a TKO orthotopic tumor, a TKO intravesical tumor, and a MB49 intravesical tumor. Orthotopic injection was performed by bladder wall injection with Hamilton microliter syringe, whereas intravesical injection by a 24G catheter delivery. (**D**) H&E staining of TKO orthotopic tumor and TKO intravesical tumor. (**E**) A TKO orthotopic tumor showed muscle-invasive disease.

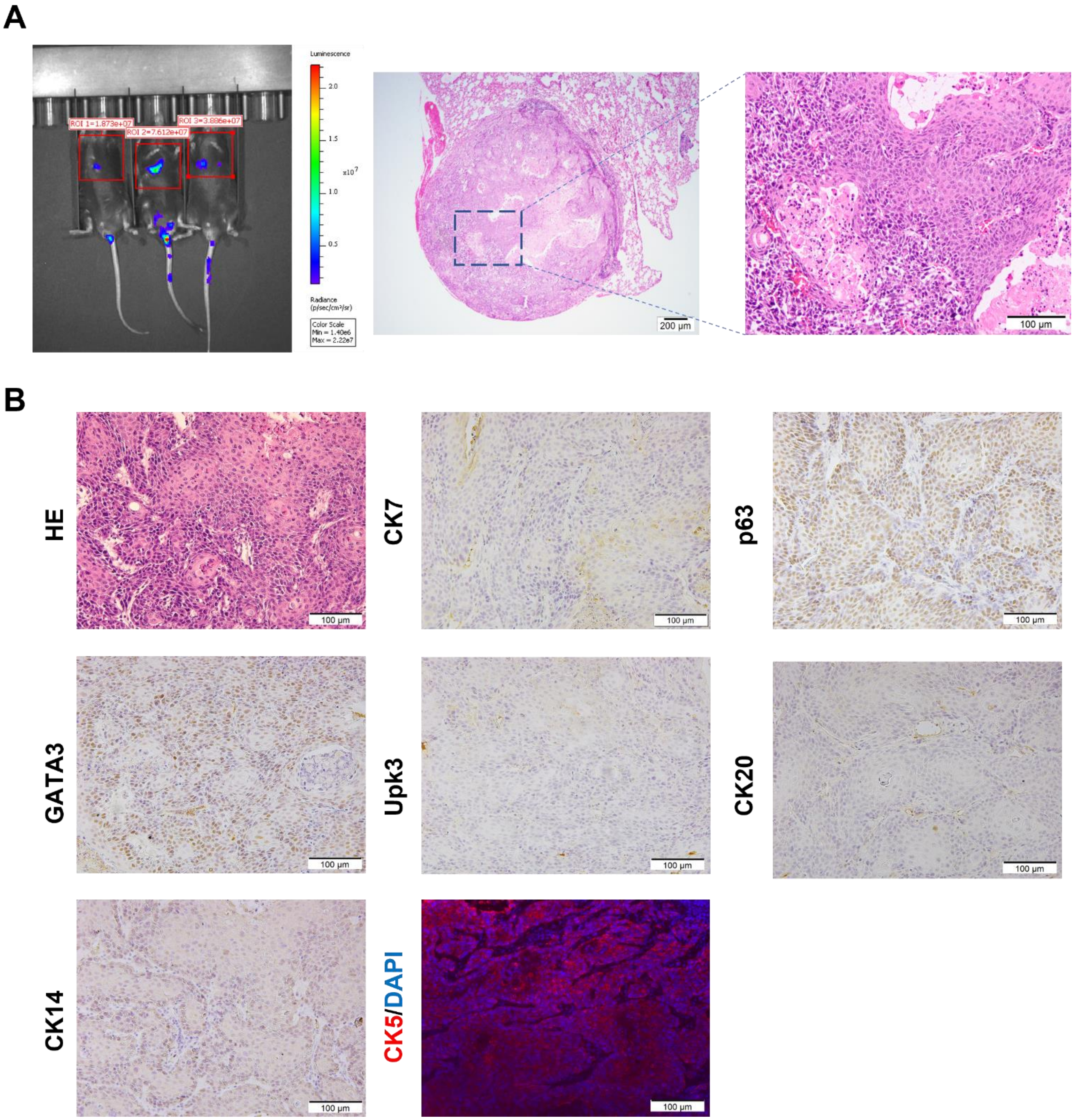

Supplemental Figure S3. The tumor formation of the TKO cells by tail vein injections and bladder orthotopic injections (into the bladder wall) in C57 BL/6J mice. (**A**) The TKO cells were labeled by *pFUGW-Pol2-ffLuc2-eGFP* (Addgene: Plasmid #71394). 1 million TKO cells with luciferase tag were injected into mouse tail vein of C57 BL/6J mice. Luminescence was detected on day 29 after injection in all (3/3) mice. H&E staining of lung tumor nodule at the endpoint of day 29. (**B**) H&E and IHC staining of indicated markers in the TKO orthotopic bladder tumors.

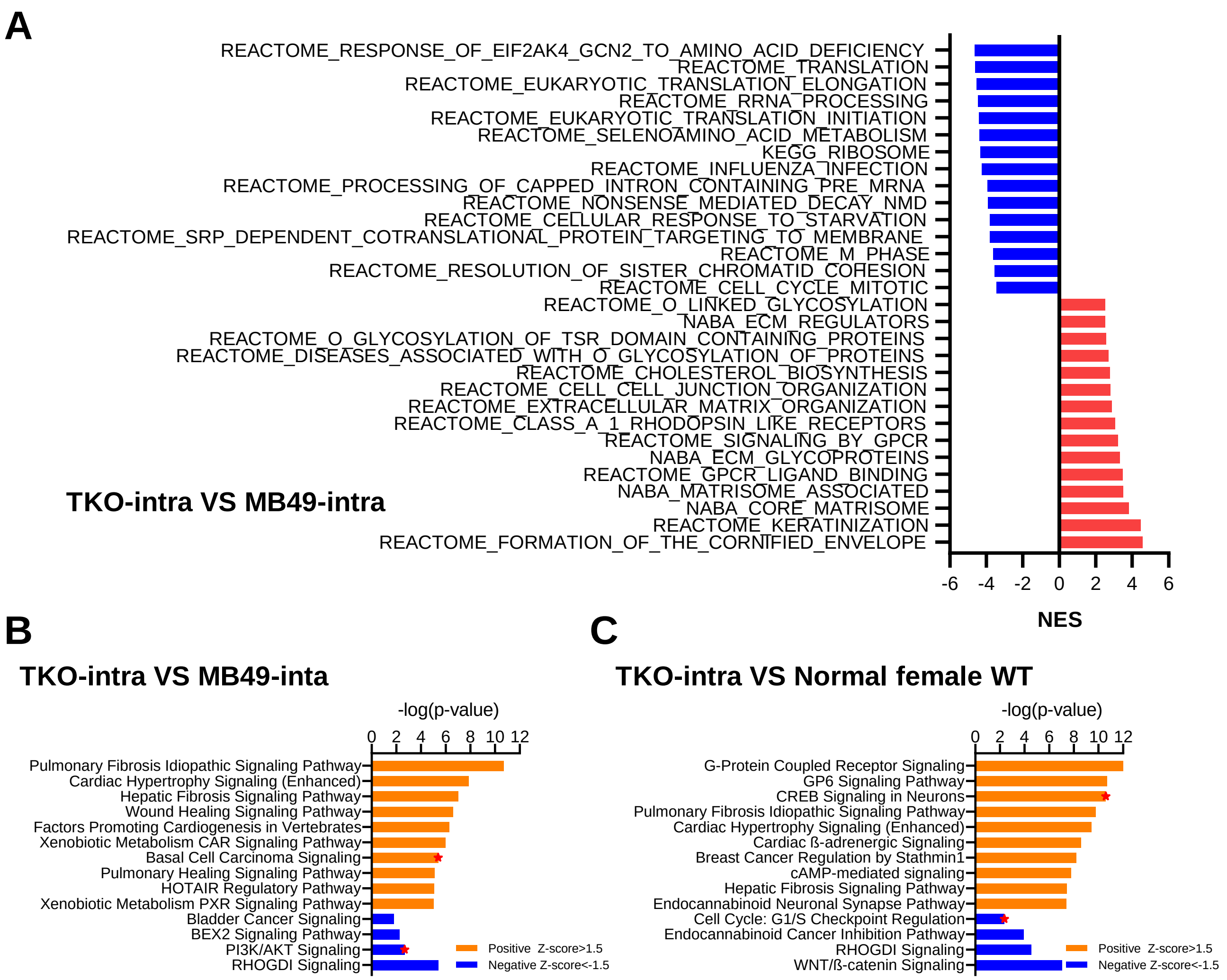

Supplemental Figure S4. GSEA analysis and IPA analysis of TKO intravesical tumors. (**A**) GSEA analysis comparing TKO-intra tumors and MB49-intra tumors. Positive normalized enrichment scores (NES) in red color indicated up-regulation in TKO-intra, whereas negative NES score in blue color indicated down-regulation in TKO-intra tumor. (**B**) Top significantly affected (Z score > 1.5 or < -1.5) canonical pathways based on IPA analysis comparing TKO-intra and MB49-intra tumors samples. Yellow color indicates activation, while blue color indicates inhibition. (**C**) Canonical pathways analysis by IPA comparing TKO-intra and normal female wild-type (WT) urothelial cells.

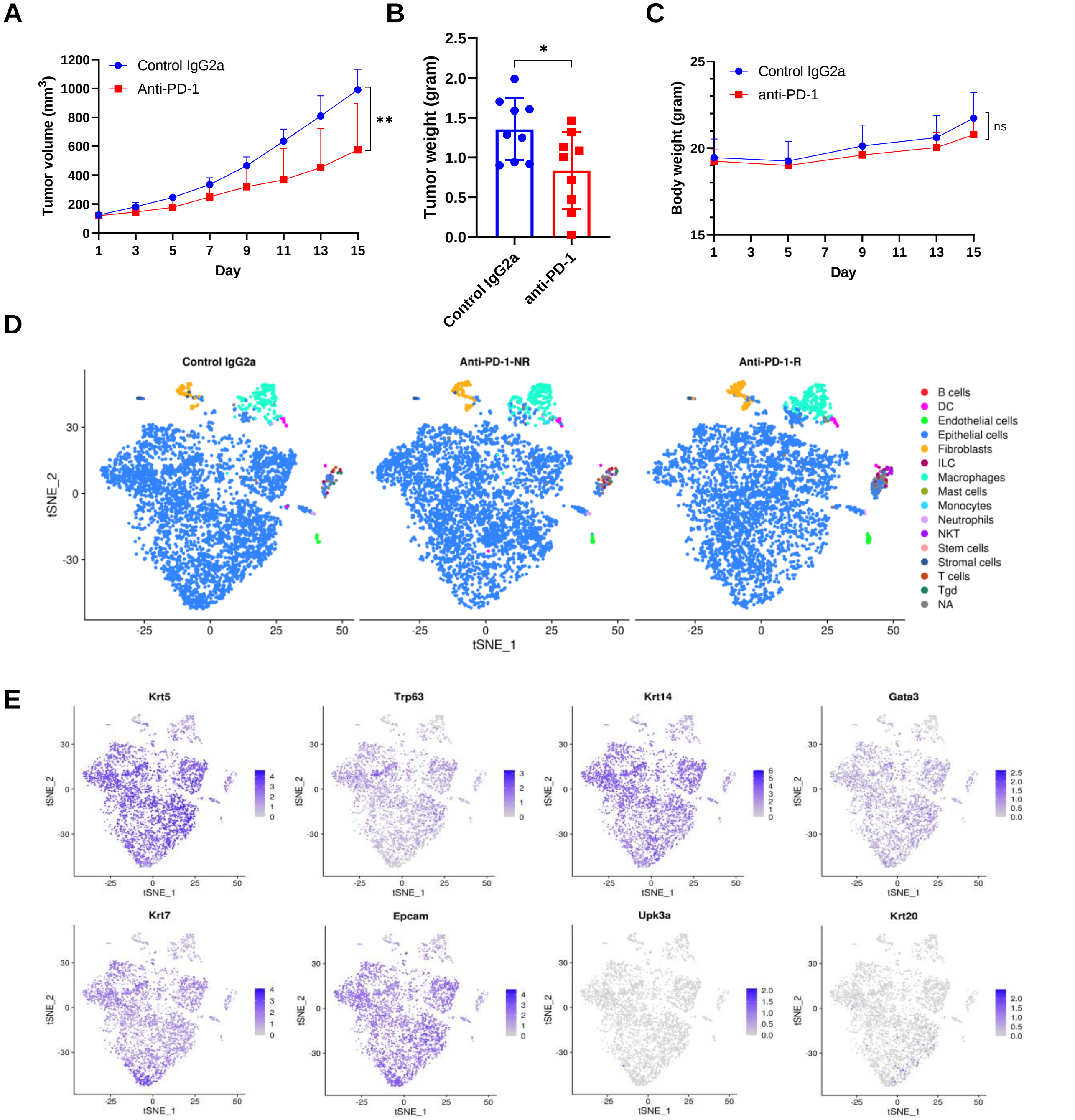

Supplemental Figure S5. Anti-PD-1 treatment of TKO subcutaneous tumors in C57 BL/6J mice and single cell analysis. (**A**) Mean tumor volume changes in C57 mice bearing TKO tumors treated with anti-PD-1 (n = 9, red) or a control IgG2a (n = 9, blue). (**B**) Mean tumor weights of TKO tumors treated with anti-PD-1 (n = 9, red) or a control IgG2a (n = 9, blue). (**C**) Mean body weight changes in C57 mice treated with anti-PD-1 and control IgG2a. (**D**) t-SNE clustering analyses of all cells in xenograft tumors from control IgG2a treatment (n = 2, 4762), anti-PD-1 non-responders (n = 2, 4459) and anti-PD-1 responders (n = 2, 4861). (**E**) Feature single gene plots for visualizing urothelial biomarkers (Krt5, Trp63, Krt14, GATA3, Krt7, EpCAM, Upk3a and Krt20) genes expression in Control IgG2a tumors. Single gene expression pattern was confirmed the IHC staining results. *p*-value, Welch's *t*-test.

A

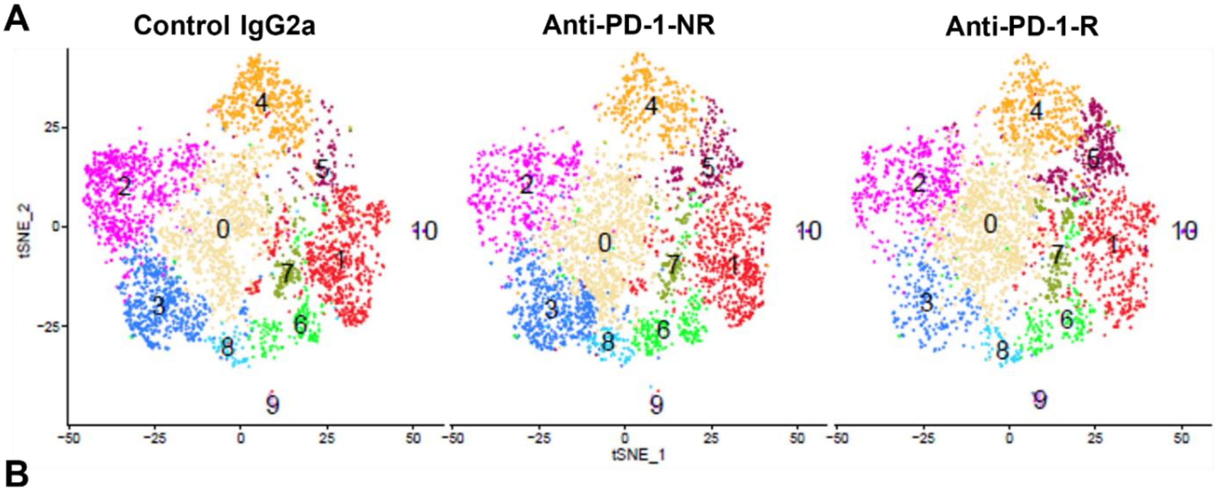

B

Cell counts and percentages of identified clusters in EpCAM highly expressed tumor cells

Cluster	Control IgG2a Cell count	Anti-PD-1-NR Cell count	Anti-PD-1-R Cell count	Control IgG2a % in tumor cell	Anti-PD-1-NR % in tumor cell	Anti-PD-1-R % in tumor cell
0	783	945	1110	18.07	24.40	28.53
1	810	722	535	18.69	18.64	13.75
2	905	491	557	20.89	12.68	14.32
3*	665	573	232	15.35	14.79	5.96
4	590	371	429	13.62	9.58	11.03
5*	106	233	488	2.45	6.02	12.54
6	212	278	211	4.89	7.18	5.42
7	156	133	196	3.60	3.43	5.04
8	73	108	67	1.68	2.79	1.72
9	16	8	48	0.37	0.21	1.23
10	17	11	18	0.39	0.28	0.46

*cluster 3 and 5 changed significantly across 3 samples.

Supplementary Figure S6. Re-analyzing EpCAM highly expressed tumor cells. (**A**) t-SNE clustering of re-analyzing EpCAM highly expressed tumor cells in the TKO tumor treated with IgG2a, anti-PD-1 (non-responders), and anti-PD-1 (responders). (**B**) Cell counts and percentages of identified clusters with EpCAM highly expression. Cluster 3 significantly decreased and cluster 5 increased after an-PD-1 treatment (*).

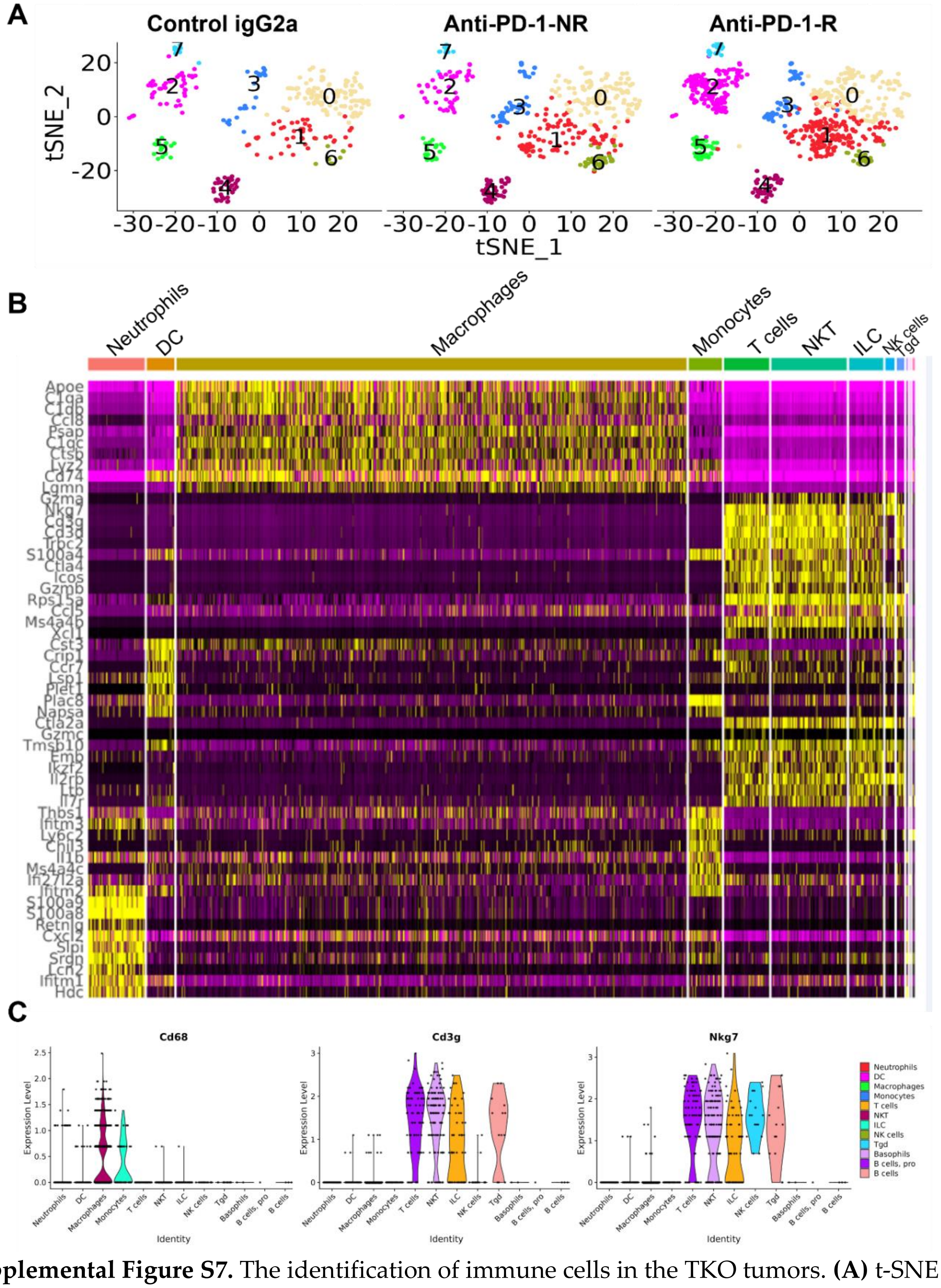

Supplemental Figure S7. The identification of immune cells in the TKO tumors. **(A)** t-SNE clustering of immune cells with high CD45 expression. Eight clusters of immune cells were identified in tumors. (**B**) Clustering heatmap of immune cell types in the TKO tumors across the top 10 most differentially expressed genes. (**C**) Violin plots of representative immune cell markers in macrophage (Cd68), T cells (Cd3g), and NKT cells (Nkg7).

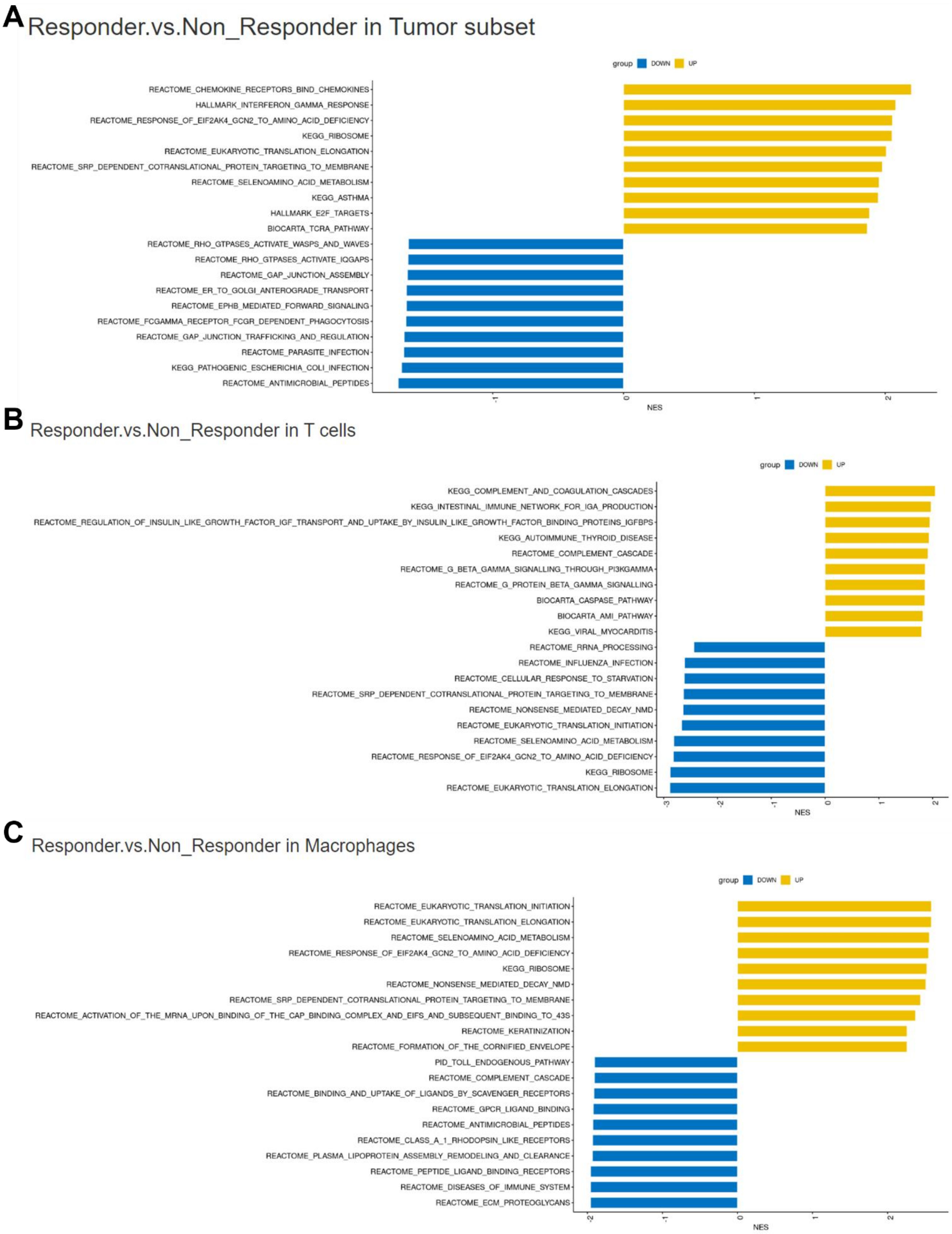
A
Responder.vs.Non_Responder in Tumor subset
group DOWN UP
REACTOME_CHEMOKINE_RECEPTORS_BIND_CHEMOKINES
HALLMARK_INTERFERON_GAMMA_RESPONSE
REACTOME_RESPONSE_OF_EIF2AK4_GCN2_TO_AMINO_ACID_DEFICIENCY
KEGG_RIBOSOME
REACTOME_EUKARYOTIC_TRANSLATION_ELONGATION
REACTOME_SRP_DEPENDENT_COTRANSLATIONAL_PROTEIN_TARGETING_TO_MEMBRANE
REACTOME_SELENOAMINO_ACID_METABOLISM
KEGG_ASTHMA
HALLMARK_E2F_TARGETS
BIOCARTA_TCRA_PATHWAY
REACTOME_RHO_GTPASES_ACTIVATE_WASPS_AND_WAVES
REACTOME_RHO_GTPASES_ACTIVATE_IQGAPS
REACTOME_GAP_JUNCTION_ASSEMBLY
REACTOME_ER_TO_GOLGI_ANTEROGRADE_TRANSPORT
REACTOME_EPHB_MEDIATED_FORWARD_SIGNALING
REACTOME_FCGAMMA_RECEPTOR_FCGR_DEPENDENT_PHAGOCYTOSIS
REACTOME_GAP_JUNCTION_TRAFFICKING_AND_REGULATION
REACTOME_PARASITE_INFECTION
KEGG_PATHOGENIC_ESCHERICHIA_COLI_INFECTION
REACTOME_ANTIMICROBIAL_PEPTIDES
-1
0
1
2
NES
B
Responder.vs.Non_Responder in T cells
group DOWN UP
KEGG_COMPLEMENT_AND_COAGULATION_CASCADES
KEGG_INTESTINAL_IMMUNE_NETWORK_FOR_IGA_PRODUCTION
REACTOME_REGULATION_OF_INSULIN_LIKE_GROWTH_FACTOR_IGF_TRANSPORT_AND_UPTAKE_BY_INSULIN_LIKE_GROWTH_FACTOR_BINDING_PROTEINS_IGFBPS
KEGG_AUTOIMMUNE_THYROID_DISEASE
REACTOME_COMPLEMENT_CASCADE
REACTOME_G_BETA_GAMMA_SIGNALLING_THROUGH_PI3KGAMMA
REACTOME_G_PROTEIN_BETA_GAMMA_SIGNALLING
BIOCARTA_CASPASE_PATHWAY
BIOCARTA_AMI_PATHWAY
KEGG_VIRAL_MYOCARDITIS
REACTOME_RRNA_PROCESSING
REACTOME_INFLUENZA_INFECTION
REACTOME_CELLULAR_RESPONSE_TO_STARVATION
REACTOME_SRP_DEPENDENT_COTRANSLATIONAL_PROTEIN_TARGETING_TO_MEMBRANE
REACTOME_NONSENSE_MEDIATED_DECAY_NMD
REACTOME_EUKARYOTIC_TRANSLATION_INITIATION
REACTOME_SELENOAMINO_ACID_METABOLISM
REACTOME_RESPONSE_OF_EIF2AK4_GCN2_TO_AMINO_ACID_DEFICIENCY
KEGG_RIBOSOME
REACTOME_EUKARYOTIC_TRANSLATION_ELONGATION
-3
-2
-1
0
1
2
NES
C
Responder.vs.Non_Responder in Macrophages
group DOWN UP
REACTOME_EUKARYOTIC_TRANSLATION_INITIATION
REACTOME_EUKARYOTIC_TRANSLATION_ELONGATION
REACTOME_SELENOAMINO_ACID_METABOLISM
REACTOME_RESPONSE_OF_EIF2AK4_GCN2_TO_AMINO_ACID_DEFICIENCY
KEGG_RIBOSOME
REACTOME_NONSENSE_MEDIATED_DECAY_NMD
REACTOME_SRP_DEPENDENT_COTRANSLATIONAL_PROTEIN_TARGETING_TO_MEMBRANE
REACTOME_ACTIVATION_OF_THE_MRNA_UPON_BINDING_OF_THE_CAP_BINDING_COMPLEX_AND_EIFS_AND_SUBSEQUENT_BINDING_TO_43S
REACTOME_KERATINIZATION
REACTOME_FORMATION_OF_THE_CORNIFIED_ENVELOPE
PID_TOLL_ENDOGENOUS_PATHWAY
REACTOME_COMPLEMENT_CASCADE
REACTOME_BINDING_AND_UPTAKE_OF_LIGANDS_BY_SCAVENGER_RECEPTORS
REACTOME_GPCR_LIGAND_BINDING
REACTOME_ANTIMICROBIAL_PEPTIDES
REACTOME_CLASS_A_1_RHODOPSIN_LIKE_RECEPTORS
REACTOME_PLASMA_LIPOPROTEIN_ASSEMBLY_REMODELING_AND_CLEARANCE
REACTOME_PEPTIDE_LIGAND_BINDING_RECEPTORS
REACTOME_DISEASES_OF_IMMUNE_SYSTEM
REACTOME_ECM_PROTEOGLYCANS
-2
-1
0
1
2
NES

Supplemental Figure S8. The Gene Set Enrichment Analysis of tumor cells and immune cells in responders and non-responders. The significant pathways (*padj* < 0.05) were ranked by NES (Normalized Enrichment Scores). Positive NES score in yellow color indicated up-regulation in responders, whereas negative NES score in blue color indicated down-regulation in responders. **(A)** Top significantly affected pathways of comparing tumor cells in responders and tumor cells in non-responders. Note chemokine receptor and IFN-gamma response related genes are upregulated in tumor cells responders compared to tumor cells in non-responders. **(B)** Top significantly affected pathways of comparing T cells in responders and T cells in non-responders. **(C)** Top significantly affected pathways of comparing macrophages in responders and macrophages in non-responders.

Supplemental Table S1. The primers used in this study for genotyping.

**Primer**	**Sequence 5′ to 3′**
Trp53 10F5	GTTAAGGGGTATGAGGGACAAGGTA
Trp53 102.3	CCATGAGACAGGGTCTTGCTATTGT
Trp53 1F5	GTGCCCTCCGTCCTTTTTCGCAATC
Rb1 18M3	GGAATTCCGGCGTGTGCCATCAATG
Rb1 19EM5	AGCTCTCAAGAGCTCAGACTCATGG
Rb1 212M5	CGAAAGGAAAGTCAGGGACATTGGG
Pten genotype-F	CAAGCACTCTGCGAACTGAG
Pten genotype-R	AAGTTTTTGAAGGCAAGATGC
Pten Del-R	GCTTGATATCGAATTCCTGCAGC

Supplemental Table S2. The antibodies used in this study.

Antibody	Company	Catalog	Applications
Monoclonal mouse anti-CK20	DAKO	M7019	IHC 1:100/IF 1:100
Monoclonal mouse anti-CK7	Santa Cruz Biotechnology	SC-23876	IHC 1:50/IF 1:50
Monoclonal mouse anti-p63	Abcam	ab735	IHC 1:100/IF 1:50
Monoclonal mouse anti-Upk3	Fitzgerald	10R-U103A	IHC 1:50
Monoclonal rat anti-CK8	Developmental Studies Hybridoma Bank	TROMA-I-s	IF 1:100
Polyclonal chicken anti-CK5	Biolegend	905901	IF 1:500
Monoclonal mouse anti-GATA3	Santa Cruz Biotechnology	SC-268	IHC 1:200
Monoclonal mouse anti-CK14	Invitrogen	MA5-11599	IHC 1:150
Monoclonal rat anti-CD4	Invitrogen	14-9766-82	IHC 1:250
Monoclonal rabbit anti-CD8	Cell Signaling Technology	98941S	IHC 1:400
Monoclonal rabbit anti-PD-1	Cell Signaling Technology	D7D5W	IHC 1:200
Monoclonal rat anti-F4/80	Biolegend	123101	IHC 1:600
Goat anti-Rat IgG, Alexa Fluor 488	Invitrogen	A-11006	IF 1:250
Goat anti-Chicken IgG, Alexa Fluor 594	Invitrogen	A-11042	IF 1:1000
Goat anti-Mouse IgG, Alexa Fluor 488	Invitrogen	A-11001	IF 1:250
Goat anti-Mouse IgG, Alexa Fluor 594	Invitrogen	A-11005	IF 1:1000

Supplemental Table S3. The cell counts and percentages of identified cell types in all cells.

Cell type	Control IgG2a Cell count	Anti-PD-1-NR Cell count	Anti-PD-1-R Cell count	Control IgG2a % in total cell	Anti-PD-1-NR % in total cell	Anti-PD-1-R % in total cell
B cells	0	0	2	0.00	0.00	0.04
DC	16	17	25	0.34	0.38	0.51
Endothelial cells	20	16	39	0.42	0.36	0.80
Epithelial cells	4398	3990	4147	92.36	89.48	85.31
Fibroblasts	75	97	140	1.57	2.18	2.88
ILC	7	7	39	0.15	0.16	0.80
Macrophages	164	236	285	3.44	5.29	5.86
Mast cells	0	0	1	0.00	0.00	0.02
Monocytes	7	11	11	0.15	0.25	0.23
Neutrophils	5	15	3	0.10	0.34	0.06
NKT	2	2	16	0.04	0.04	0.33
Stem cells	1	0	0	0.02	0.00	0.00
Stromal cells	20	8	20	0.42	0.18	0.41
T cells	4	9	21	0.08	0.20	0.43
Tgd	3	4	5	0.06	0.09	0.10
NA	40	47	107	0.84	1.05	2.20
